# The method of detection of ductal carcinoma in situ has no therapeutic implications: results of a population-based cohort study

**DOI:** 10.1186/s13058-017-0819-4

**Published:** 2017-03-09

**Authors:** Lotte E. Elshof, Michael Schaapveld, Emiel J. Rutgers, Marjanka K. Schmidt, Linda de Munck, Flora E. van Leeuwen, Jelle Wesseling

**Affiliations:** 1 Division of Molecular Pathology, Netherlands Cancer Institute/Antoni van Leeuwenhoek, Plesmanlaan 121, 1066 CX Amsterdam, The Netherlands; 2 Division of Psychosocial research and Epidemiology, Netherlands Cancer Institute/Antoni van Leeuwenhoek, Plesmanlaan 121, 1066 CX Amsterdam, The Netherlands; 3 Department of Surgery, Netherlands Cancer Institute/Antoni van Leeuwenhoek, Plesmanlaan 121, 1066 CX Amsterdam, The Netherlands; 4 Department of Research, Netherlands Comprehensive Cancer Organisation (IKNL), Godebaldkwartier 419, 3511 DT Utrecht, The Netherlands; 5 Department of Pathology, Netherlands Cancer Institute/Antoni van Leeuwenhoek, Plesmanlaan 121, 1066 CX Amsterdam, The Netherlands

**Keywords:** Ductal carcinoma in situ, Invasive breast cancer, Mortality, Screening, Population-based cohort, Detection, Mammography

## Abstract

**Background:**

Population screening with mammography has resulted in increased detection of ductal carcinoma in situ (DCIS). The aim of this population-based cohort study was to assess whether the method of detection should be considered when determining prognosis and treatment in women with DCIS.

**Methods:**

This study includes 7042 women aged 49–75 years, who were surgically treated for primary DCIS between 1989 and 2004 in the Netherlands. We calculated cumulative incidences of ipsilateral and contralateral invasive breast cancer and all-cause mortality among women with screen-detected, interval, or non-screening-related DCIS, and assessed the association between method of detection and these outcomes, using multivariable Cox regression analyses.

**Results:**

Compared with non-screening-related DCIS, women with screen-detected DCIS had a lower risk of developing ipsilateral invasive breast cancer (hazard ratio (HR) = 0.75, 95% CI = 0.59–0.96), but a similar risk of contralateral invasive breast cancer (HR = 0.86, 95% CI = 0.67–1.10). The absolute difference in risk of ipsilateral invasive breast cancer was 1% at 15 years. Screen detection was associated with lower all-cause mortality (HR = 0.85, 95% CI = 0.73–0.98); when we additionally accounted for the occurrence of invasive breast cancer the magnitude of this effect remained similar (HR = 0.86, 95% CI = 0.75–1.00).

**Conclusions:**

Screen detection was associated with lower risk of ipsilateral invasive breast cancer and all-cause mortality. However, the absolute difference in risk of ipsilateral invasive breast cancer was very low and the lower all-cause mortality associated with screen-detected and interval DCIS might be explained by a healthy-user effect. Therefore, our findings do not justify different treatment strategies for women with screen-detected, interval, or non-screening-related DCIS.

**Electronic supplementary material:**

The online version of this article (doi:10.1186/s13058-017-0819-4) contains supplementary material, which is available to authorized users.

## Background

Population-based breast cancer screening has been introduced on the basis of evidence that mammographic screening could reduce mortality in breast cancer. Yet, there is an ongoing debate about one of the major concerns of screening, overdiagnosis, which is the detection of an abnormality that would never have caused symptoms or death during one’s lifetime if screening had been omitted [1–7]. Unfortunately, we are not able to distinguish between women who are overdiagnosed and women who do need treatment. Therefore, we tend to treat them all. This implies that overdiagnosis results in overtreatment [8].

Population screening with mammography has resulted in increased detection of ductal carcinoma in situ (DCIS), a non-obligate precursor lesion for invasive breast cancer. Several studies have indicated an association between mammographic screening and overdiagnosis and overtreatment of DCIS [9–12]. On the other hand, a recent ecological study reported that a higher rate of screen-detected DCIS is associated with a lower rate of invasive interval cancers, suggesting that detection and treatment of DCIS is worthwhile [13].

Method of detection has been shown to be an independent prognostic factor beyond stage migration in patients with invasive breast carcinoma [14, 15]. Women with invasive breast cancer detected at population-based screening have been shown to have better overall and breast cancer-specific survival than those who have not participated in the screening program, with an absolute reduction in breast cancer-specific mortality of 7% at 10 years [14].

Previous studies suggest that the method of detection may also carry prognostic information in women treated for DCIS [16–19], but it is still unclear whether women with screen-detected DCIS have a clinically relevant better prognosis than women with non-screening-related DCIS and whether the method for detection should be used in the treatment decision-making process, such as the addition or omission of radiotherapy and anti-estrogen treatment. To this end, we studied risk of subsequent ipsilateral and contralateral invasive breast cancer and all-cause mortality among a large population-based cohort of women with screen-detected, interval, and non-screening-related DCIS.

## Methods

### Patient selection

All women who were diagnosed with DCIS in the Netherlands from 1989 through 2004, and who had no previous malignancies except for non-melanoma skin cancer, were selected from a cohort based on linked data from the Netherlands Cancer Registry (NCR) and the nationwide network and registry of histology and cytopathology in the Netherlands (PALGA) [20]. To be eligible for the current study we required: (1) a diagnosis of pure DCIS; (2) age 49–75 years at DCIS diagnosis; (3) DCIS treatment consisting of breast-conserving surgery plus radiotherapy, breast-conserving surgery alone, or mastectomy; and (4) no invasive breast cancer or second breast carcinoma in situ within 4 months after initial DCIS diagnosis. Women who were diagnosed at autopsy (*n* = 1), women in whom the type of surgery could not be determined (*n* = 59), and women who received chemotherapy or hormonal therapy for DCIS (*n* = 47) were excluded.

### Breast cancer screening program

The Dutch breast cancer screening program started in 1989 [21]. From 1989 to 1997 women aged 50–69 years were the target population. Full coverage of these women was achieved in 1997 [22, 23]. In 1998 the program was extended to women aged 70–75 years. In the Dutch screening program women receive an invitation for screening mammography once every 2 years starting in the year the women turn 50 or 51 years. Invitations for the next round are issued within 24 + 2 months of the prior screening. For women who move to another local authority area the next screening could be delayed up to 6 months. Screening mammograms are performed in independent and mostly mobile screening units, and the images are interpreted double-blinded by trained radiologists. Information about screening mammography is recorded by the five regional screening facilities and collected in the database of the Dutch breast cancer screening organization.

### Method of detection

To obtain information about the method of detection we linked our dataset with the database of the Dutch breast cancer screening organization. We classified three categories of DCIS on the basis of method of detection: (1) screen-detected DCIS, defined as DCIS that was detected <12 months after a first or subsequent positive screening examination in the Dutch breast cancer screening program; (2) interval DCIS, defined as DCIS diagnosed <30 months after a negative screening examination, or diagnosed 12–30 months after a positive screening examination; and (3) DCIS detected outside the breast cancer screening program (non-screening-related), defined as DCIS diagnosed ≥30 months after the screening examination, diagnosed prior to the first screening examination, or diagnosed in women who never participated in the program.

Women who had ever participated in the screening program, but for whom method of DCIS detection was unknown, were excluded from the analyses (*n* = 525). To examine possible confounding by excluding this group of women, we compared the risk of subsequent ipsilateral and contralateral invasive breast cancer and all-cause mortality between this group and the group of women with a known method of detection: no significant differences were found. Our study cohort included 7042 women in whom the method of detection was known.

Because of the gradual implementation of the screening program in the Netherlands, we defined two periods of DCIS diagnosis, 1989–1998 and 1999–2004. In the first period the women with non-screening-related DCIS were more likely not to be invited for breast cancer screening, while in the later period, the group of women with non-screening-related DCIS could have comprised more women who chose not to participate in breast cancer screening.

### Treatment and grade

Information about surgical DCIS treatment was obtained from the NCR database and further completed based on PALGA data. Further, the NCR provided information on whether radiotherapy was administered. Initial DCIS treatment was defined as the treatment strategy for the primary DCIS lesion within 3 months of diagnosis. Three categories were classified: breast-conserving surgery plus radiotherapy, breast-conserving surgery alone, and mastectomy. Additionally, using PALGA data we assessed whether women initially treated by breast-conserving surgery had undergone ipsilateral mastectomy during follow up (due to any cause other than ipsilateral invasive breast cancer). Information on grade was derived from the NCR and was available for 56.4% of the analytical cohort. The grading system used in the Netherlands is based on the classification presented by Holland et al. [24].

### Outcome data and statistical analyses

Follow up of ipsilateral and contralateral invasive breast cancer and vital status was obtained from the NCR and PALGA databases and was complete up to 1 January 2011. The period of time at risk started at the date of DCIS diagnosis, and stopped at the date of the event of interest, emigration, or 31 December 2010, whichever came first.

Up to 15-year cumulative incidences of ipsilateral and contralateral invasive breast cancer were calculated according to method of detection, using death as a competing risk. All-cause mortality was calculated according to method of detection and age group at DCIS diagnosis using the Kaplan-Meier method. *P* values were based either on competing risk regression [25] or Cox proportional hazards regression, with time since DCIS diagnosis as the time scale and adjusted for age (continuous).

We used multivariable-adjusted Cox proportional hazards analyses to estimate relative differences in risk of ipsilateral and contralateral invasive breast cancer and all-cause mortality among women with screen-detected, interval, and non-screening-related DCIS. In these analyses we used age as the primary time scale, and time since DCIS diagnosis (0–5, 5–10, and ≥10 years) as secondary time scale. We adjusted for DCIS treatment (time-varying), DCIS grade and period of diagnosis.

We performed a supplementary analysis restricted to women participating in the implemented Dutch screening program, in which we compared risk of ipsilateral and contralateral invasive breast cancer and mortality among women with screen-detected vs. interval DCIS between 1999 and 2004.

Data on cause of death were not available in this study. In an attempt to correct for death due to invasive breast cancer we additionally included the occurrence of ipsilateral and contralateral invasive breast cancer as time-varying covariables in the model, with all-cause mortality as outcome.

The proportional hazard assumption was verified using graphical and residual-based methods. Furthermore, we assessed whether the effect of method of detection was modified by period of diagnosis by introducing an appropriate interaction term in the model.

A *P* value <0.05 was considered statistically significant. All analyses were performed with STATA/SE 13.1 (StataCorp LP, College Station, TX, USA). The study was approved by the review boards of the NCR, PALGA, and the Dutch breast cancer screening organization.

## Results

### Study population and method of detection

The study cohort comprised 4814 women with screen-detected DCIS, 651 with interval DCIS, and 1577 with non-screening-related DCIS. Among the women with screen-detected DCIS, 1622 (34%) were detected in the first screening round (i.e. prevalent DCIS), and 3192 (66%) in a subsequent screening round (i.e. incident DCIS).

**Fig. 1:**
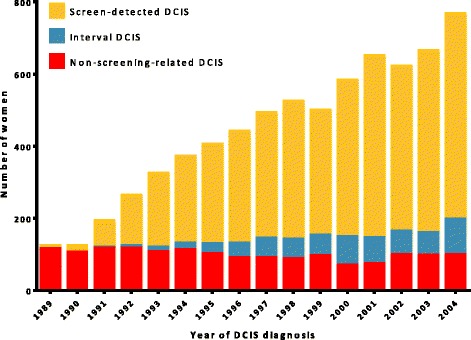
Method of detection by year of diagnosis of ductal carcinoma in situ (*DCIS*)

Most screen-detected DCIS was diagnosed within 2 months after the screening mammogram was performed (90th percentile) (Additional file 1). The proportion of screen-detected DCIS increased over time (*P* < 0.001), whereas the absolute number of non-screening-related DCIS remained stable (Fig. 1). The distribution of grade was dependent on screening status (*P* < 0.001) (Table 1). Of the women with screen-detected DCIS, 59% were treated by breast-conserving surgery, compared to 51% of interval DCIS, and 46% of non-screening-related DCIS (Table 1). The proportion of women undergoing mastectomy decreased over time in all detection groups (*P* < 0.001).

**Table 1:** Characteristics of the study population by method of detection

Method of detection	Non-screening-related	Screen-detected	Interval
DCIS diagnosis			
Within 12 months after positive first screening, *n* (%)	NA	1622 (33.7)	NA
Within 12 months after positive subsequent screening, *n* (%)	NA	3192 (66.3)	NA
Within 30 months of negative screening, *n* (%)	NA	NA	577 (88.6)
12 to 30 months after positive screening, *n* (%)	NA	NA	74 (11.4)
≥30 months after screening participation, *n* (%)	159 (10.1)	NA	NA
Prior to first screening participation, *n* (%)	115 (7.3)	NA	NA
In women who never participated in the screening program, *n* (%)	1303 (82.6)	NA	NA
Mammography type^a^			
Conventional (film)	159	4804	651
Digital	0	10	0
Age at DCIS diagnosis, years			
Median (interquartile range)	58.1 (51.9–66.8)	58.7 (53.5–64.3)	59.8 (55.0–65.7)
49–59, *n* (%)	868 (55.0)	2676 (55.6)	327 (50.2)
60–69, *n* (%)	450 (28.5)	1789 (37.2)	262 (40.3)
70–75, *n* (%)	259 (16.4)	349 (7.3)	62 (9.5)
Period of DCIS diagnosis			
1989–1998 (implementation phase), *n* (%)	1044 (66.2)	2001 (41.6)	215 (33.0)
1999–2004 (full nationwide coverage), *n* (%)	533 (33.8)	2813 (58.4)	436 (67.0)
DCIS grade			
1	126 (8.0)	440 (9.1)	90 (13.8)
2	188 (11.9)	926 (19.2)	127 (19.5)
3	288 (18.3)	1577 (32.8)	208 (32.0)
Unknown^b^	975 (61.8)	1871 (38.9)	226 (34.7)
DCIS treatment within 3 months of diagnosis			
Breast-conserving surgery with radiotherapy, *n* (%)	276 (17.5)	1587 (33.0)	188 (28.9)
Breast-conserving surgery without radiotherapy, *n* (%)	443 (28.1)	1256 (26.1)	144 (22.1)
Mastectomy, *n* (%)	858 (54.4)	1971 (40.9)	319 (49.0)
Follow-up interval, years			
Median (interquartile range)	12.2 (8.1–16.4)	10.3 (7.7–13.5)	9.9 (7.3–12.5)
0–4^c^, *n* (%)	115 (7.3)	179 (3.7)	29 (4.5)
5–9, *n* (%)	461 (29.2)	2117 (44.0)	305 (46.9)
≥10, *n* (%)	1001 (63.5)	2518 (52.3)	317 (48.7)
Subsequent invasive breast cancer^d^			
No, *n* (%)	1385 (87.8)	4357 (90.5)	592 (90.9)
Ipsilateral only, *n* (%)	87 (5.5)	213 (4.4)	29 (4.5)
Contralateral only, *n* (%)	94 (6.0)	225 (4.7)	25 (3.8)
Ipsilateral + contralateral, *n* (%)	11 (0.7)	18 (0.4)	5 (0.8)
Vital status at last follow up			
Alive, *n* (%)	1153 (73.1)	4191 (87.1)	575 (88.3)
Dead, *n* (%)	398 (25.2)	594 (12.3)	68 (10.5)
Emigrated, *n* (%)	26 (1.7)	29 (0.6)	8 (1.2)
Total	1577	4814	651

^a^Last screening mammogram before diagnosis of ductal carcinoma in situ (DCIS). ^b^1989–1998: 75% unknown vs. 1999–2004: 16% unknown. ^c^Eight patients with follow-up time = 0. ^d^One patient with unknown laterality of subsequent invasive breast cancer. *NA* not applicable

### Ipsilateral and contralateral invasive breast cancer

**Fig. 2:**
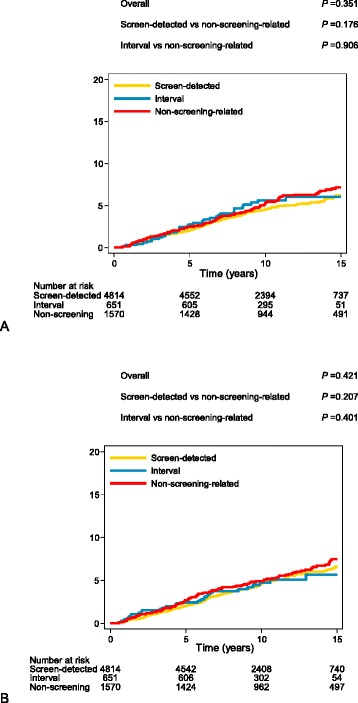
Cumulative incidence of ipsilateral (**a**) and contralateral (**b**) invasive breast cancer by method of detection, with death analyzed as a competing risk. *P* values based on competing risk regression with time since diagnosis of ductal carcinoma in situ as the primary time scale, adjusted for age (continuous) [25]

With a median follow up of 10.5 years (interquartile range = 7.7–14.0), 363 and 378 of 7042 women were diagnosed with ipsilateral and contralateral invasive breast cancer, respectively. Women with screen-detected DCIS had lower risk of ipsilateral invasive breast cancer than women with non-screening-related DCIS (adjusted hazard ratio (HR) = 0.75, 95% confidence interval (CI) = 0.59–0.96) (Table 2, Additional file 2). The absolute difference in risk of ipsilateral invasive breast cancer at 15 years of follow up, between screen-detected and non-screening-related DCIS was 1% (cumulative incidence = 6% vs. 7%, respectively) (Fig. 2). No statistically significant difference was observed between interval and non-screening-related DCIS (HR = 1.02, 95% CI = 0.68–1.51).

**Table 2:** Multivariable-adjusted Cox regression analyses for different events in women aged 49–75 years at DCIS diagnosis

Method of detection	Total number of events	Person-time, years	HR (95% CI)	*P* value
	Ipsilateral invasive breast cancer			
Non-screening-related	98	18,710	ref	
Screen-detected	231	50,422	0.75 (0.59–0.96)	0.024
Interval	34	6359	1.02 (0.68–1.51)	0.941
	Contralateral invasive breast cancer			
Non-screening-related	105	18,825	ref	
Screen-detected	243	50,467	0.86 (0.67–1.10)	0.224
Interval	30	6387	0.83 (0.54–1.26)	0.382
	All-cause mortality			
Non-screening-related	398	19,361	ref	
Screen-detected	594	51,740	0.85 (0.73–0.98)	0.028
Interval	68	6570	0.73 (0.56 − 0.96)	0.025

Analysis was performed with age as the primary time scale and time since DCIS diagnosis (0–5, 5–10, and ≥10 years) as the secondary time scale. We adjusted for period of ductal carcinoma in situ (DCIS) diagnosis, DCIS grade and DCIS treatment (time-varying). *HR* hazard ratio, *CI* confidence interval

Risk of contralateral invasive breast cancer was not associated with method of detection (screen-detected vs. non-screening-related DCIS: HR = 0.86, 95% CI = 0.67–1.10; interval versus non-screening-related DCIS: HR = 0.83, 95% CI = 0.54–1.26) (Table 2, Additional file 2). During the 15-year follow-up period, the cumulative incidence of contralateral invasive breast cancer was similar to the cumulative incidence of ipsilateral invasive breast cancer (Fig. 2). The association between method of detection and risk of ipsilateral invasive breast cancer did not differ by period of diagnosis (*P*_interaction_ = 0.540), nor did it for contralateral invasive breast cancer risk (*P*_interaction_ = 0.282).

### All-cause mortality

**Fig. 3:**
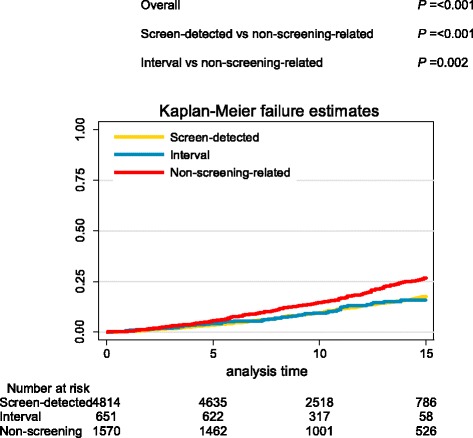
Kaplan-Meier curves for all-cause mortality by method of detection. *P* values based on Cox proportional hazards regression with time since diagnosis of ductal carcinoma in situ as the primary time scale, adjusted for age (continuous)

During follow up, 1060 of 7042 women died. In a multivariable-adjusted model, adjusted for treatment, grade and period of diagnosis, having a screen-detected or interval DCIS was associated with lower all-cause mortality compared to non-screening-related DCIS (for screen-detected DCIS, HR = 0.85, 95% CI = 0.73–0.98; for interval DCIS, HR = 0.73, 95% CI = 0.56–0.96) (Table 2, Additional file 3). Additional adjustment for the occurrence of invasive breast cancer did not affect these risk estimates, while the confidence interval changed only slightly (HR = 0.86, 95% CI = 0.75–1.00 for screen-detected DCIS; HR = 0.73, 95% CI = 0.56–0.96 for interval DCIS). The association between method of detection and all-cause mortality did not change with period of diagnosis (*P*_interaction_ = 0.531). Figure 3 shows the Kaplan-Meier curves for all-cause mortality by method of detection; there was no difference between screen-detected and interval DCIS. Differences in all cause-mortality between women attending screening (with screen-detected or interval DCIS) and women with non-screening-related DCIS were only detected in women aged 60–69 years (*P* < 0.001) (Additional file 4).

In the supplementary analysis, we found that women with interval DCIS tended to have higher risk of ipsilateral invasive breast cancer than women with screen-detected DCIS (HR 1.64; 95% CI 0.99–2.72), whereas the risk of contralateral invasive breast cancer was similar (HR 1.01; 95% CI 0.61–1.67) (Additional file 5). In addition, the risk of dying was similar in women with screen-detected DCIS and women with interval DCIS (HR 1.00; 95% CI 0.70–1.41) (Additional file 6).

## Discussion

This large nationwide cohort provides a unique opportunity to assess whether women with screen-detected DCIS have a clinically relevant better prognosis than women with non-screening-related DCIS and whether the method of detection should be used in the treatment decision-making process. We found that women with screen-detected DCIS had lower risk of subsequent ipsilateral invasive breast cancer, irrespective of age and treatment, compared to women with DCIS that was not detected within the national screening program. However, the absolute difference in risk of ipsilateral invasive breast cancer was very small; 6% of patients with screen-detected DCIS and 7% of patients with non-screening-related DCIS had developed ipsilateral invasive breast cancer at 15 years.

Further, we observed that women with screen-detected and interval DCIS had lower all-cause mortality compared to women with non-screening-related DCIS. Time-dependent adjustment for invasive breast cancer did not change the effect estimates, suggesting that the difference is caused by death due to causes other than invasive breast cancer. Importantly, this is circumstantial evidence and we cannot rule out that the difference may be partly explained by breast cancer mortality. However, breast cancer-specific mortality in women with DCIS is very low [26]. Therefore, if the difference in all-cause mortality is partly caused by breast cancer mortality, the absolute difference in breast cancer mortality between screen-detected, interval, and non-screening-related DCIS will likely be very small and clinically not significant.

Another possible explanation for lower all-cause mortality in women who participate in mammographic screening is the healthy-user effect [27]. The healthy-user effect occurs when patients who choose to receive one preventive service also choose to receive other preventive services. These patients also may have less comorbidity and better functional status, increasing the likelihood of other healthy behaviors. This is supported by a Dutch study, in which the attendance rate for breast cancer screening was lower among women with a low socioeconomic status, and comorbidity was inversely associated with socioeconomic status [28]. A healthy-user effect among women who attend mammographic screening is also supported by other studies [29–32]. Based on the available evidence, the healthy-user effect might explain the better overall survival in women with screen-detected DCIS.

In earlier studies the association between method of detection and the development of subsequent invasive breast cancer was tested in patients with DCIS, but a recent meta-analysis showed that the classification of detection method varied and had not been uniformly defined [16]. As a consequence, interpreting the association between method of detection and outcome of treatment of DCIS is more difficult. A few studies have evaluated whether women with DCIS detected through a population-based screening program have better prognosis than women with non-screening-related DCIS [18, 19], and therefore can be compared to our findings. Cheung et al. [19] (*n* = 3930 subjects, length of follow up not reported) found that the difference in risk of ipsilateral invasive breast cancer between screen-detected and non-screening-related DCIS was somewhat larger than in our study (annual absolute risk 0.43% vs. 0.65%; HR = 0.32, *P* value <0.0001), possibly because of variation in selection criteria. Our study only included women who were eligible for participation in the population-based screening program based on age, whereas Cheung et al. analyzed all women, and thus also included younger women (<50 years) who have a higher risk of invasive breast cancer compared to older women [33]. Falk et al. [18] (*n* = 3163 subjects, median follow up 5.2 years) reported a lower risk of ipsilateral invasive breast cancer in screen-detected DCIS, which was comparable to our results (HR = 0.7, 95% CI = 0.4–1.1), but did not report absolute risk estimates for groups according to different methods of detection.

The meta-analysis by Zhang et al. included six studies; in five of these studies mammographically detected DCIS was compared with symptomatic or palpable DCIS [16]. Unfortunately, we had no information on whether the DCIS was detected by mammography or by clinical symptoms. In addition, the non-screening-related group might also include women who attended opportunistic screening or who were at higher risk of breast cancer and therefore attended a different screening program. However, Zhang et al. reported a higher risk of ipsilateral invasive breast cancer in symptomatic DCIS, which was comparable to the higher risk in non-screening-related DCIS in our study (HR = 1.38, 95% CI = 1.12–1.63 and HR = 1.33, 95% CI = 1.04–1.70, respectively).

It has been postulated that the difference in risk of ipsilateral invasive breast cancer between screen-detected and non-screening-related DCIS might be due to differences in the underlying biology of DCIS [34–36]. Symptomatic presentation has been shown to be associated with larger lesion size and higher risk of estrogen receptor negativity [34, 35]. Triple-negative, human epidermal growth factor receptor 2 (HER2), and basal-like phenotypes were found to be more common in symptomatic DCIS, while the luminal A phenotype was more often observed in screen-detected DCIS [35]. Although there was a larger proportion of low-grade DCIS in the screen-detected group in one study, in others there was no difference in distribution by grade [34–36]. Also in a recent Dutch study, the distribution by grade was not dependent on mass screening status (screened population in the breast cancer screening program vs. population not subjected to/participating in mass screening) [37]. In our study the distribution by grade was dependent on screening status, but the difference in risk of ipsilateral invasive breast cancer between screen-detected and non-screening-related DCIS was independent of grade. Unfortunately, there was no information available to explore other differences in the biology of DCIS.

Breast density might be another explanation for the observed lower risk of ipsilateral invasive breast cancer among women with screen-detected DCIS. As high breast density is associated with decreased mammographic accuracy and increased risk of breast cancer [38, 39], it might be hypothesized that women with screen-detected DCIS have relatively low breast density and therefore confer a lower risk of subsequent breast cancer as compared with women with interval DCIS.

The association between method of detection and mortality in women with DCIS has been studied less frequently. Our results are in line with the study by Koh et al. (*n* = 1202 subjects, median follow up 8.2 years), which showed that women with screen-detected DCIS had better overall survival than women with symptomatic DCIS [35]. The high overall survival rates (100% and 97.8% at 10 years, respectively) presented by the authors can be explained by the large proportion (45%) of women aged <50 years at diagnosis in their study.

The prognostic value of the detection method seems to be different in patients with DCIS compared to patients with invasive breast cancer. As considerably better overall survival and breast cancer-specific survival has been observed in studies of women with screen-detected invasive breast cancer compared with non-screening-related invasive breast cancer, it has been suggested that method of detection should be used when selecting patients for adjuvant systemic therapy and that withholding chemotherapy for patients with screen-detected invasive cancer could be considered [14, 15, 40]. In contrast, our results do not support treatment decision-making, such as omitting radiation after breast-conserving surgery or using adjuvant hormonal therapy [41], based on the method of detection.

A major strength of our study is that detailed information from the Dutch breast cancer screening program was available for this large population-based cohort with substantial follow up. On top of this, we had complete data on treatment type and therefore were able to adjust for this factor in the multivariable analyses. In addition, by using age as the primary time scale in these multivariable analyses we were able to correct for age in the most optimal way.

A limitation is that the non-screening-related group represents a heterogeneous group, including both women who were not invited in the screening program and women who refused to participate. However, we found that the association between method of detection and all three outcomes was similar among women diagnosed between 1989 and 1998 (most likely random selection) and 1999 and 2004 (subject to selection bias).

Another important question is to what extent our results reflect the current situation in which screen-film mammography has been replaced by full-field digital mammography in most Western breast cancer screening programs. Digital mammography seems to be associated with a higher rate of detection of DCIS [42–44]. However, in a recent Dutch study to determine whether this transition has resulted in changes in performance indicators and characteristics of screen-detected and interval cancers, there was no increased incidence of DCIS [45]. Moreover, opposing effects have been reported on the distribution of grade. Both a higher proportion of low-grade DCIS [46] and a higher proportion of high-grade DCIS [42] at digital mammography have been described. Further, others have found no differences in the distribution of grade after digital mammography compared with screen-film mammography [43, 45]. These discrepancies complicate the translation of our results to the current digital era, but also suggest that the truth lies somewhere in between. Therefore, our results are likely very relevant for patients diagnosed with DCIS today.

## Conclusions

This study showed that having screen-detected DCIS was associated with lower risk of ipsilateral invasive breast cancer, but the absolute differences in risk of ipsilateral invasive breast cancer were not clinically significant. Women with screen-detected and interval DCIS had lower all-cause mortality compared with women with non-screening-related DCIS, which might be explained by the healthy-user effect based on the available evidence. Therefore, our findings indicate a limited prognostic role of the method of detection of DCIS, and do not justify different treatment strategies for women with screen-detected, interval, or non-screening-related DCIS.

## Additional files

Additional file 1: Time (days) between a first or subsequent screening examination by the Dutch breast cancer screening program and DCIS diagnosis in women with screen-detected DCIS (DCIS diagnostic period 1989–2004) (DOCX 17 kb)
Additional file 2: Multivariable-adjusted Cox regression analysis of ipsilateral and contralateral invasive breast cancer in women aged 49–75 years at DCIS diagnosis (DCIS diagnostic period 1989–2004). Age was the primary time scale, time since DCIS diagnosis (0–5, 5–10, and ≥10 years) the secondary time scale, and DCIS treatment a time-varying covariable (DOCX 22 kb)
Additional file 3: Multivariable-adjusted Cox regression analysis of overall mortality in women aged 49–75 years at DCIS diagnosis (DCIS diagnostic period 1989–2004). Age was the primary time scale and time since DCIS diagnosis (0–5, 5–10, and ≥10 years) the secondary time scale. Model 1 was adjusted for period of DCIS diagnosis, DCIS grade, and DCIS treatment (time-varying). Model 2 was adjusted for period of DCIS diagnosis, DCIS grade, DCIS treatment (time-varying), and the occurrence of ipsilateral and contralateral invasive breast cancer (time-varying) (DOCX 21 kb)
Additional file 4: Kaplan-Meier curves for all-cause mortality by method of detection for patients aged 49–59 years at DCIS diagnosis (a), patients aged 60–69 years at DCIS diagnosis (b), and patients aged 70–75 years (c) at DCIS diagnosis (DCIS diagnostic period 1989–2004). *P* values based on Cox proportional hazards regression with time since DCIS diagnosis as the primary time scale, adjusted for age (continuous). (DOCX 52 kb)
Additional file 5: Multivariable-adjusted Cox regression analysis of ipsilateral and contralateral invasive breast cancer in women aged 49–75 years at DCIS diagnosis: comparison between screen-detected and interval DCIS (DCIS diagnostic period 1999–2004 (screening implemented)). Age was the primary time scale and time since DCIS diagnosis (0–5, 5–10, and ≥10 years) the secondary time-scale. (DOCX 21 kb)
Additional file 6: Multivariable-adjusted Cox regression analysis of overall mortality in women aged 49–75 years at DCIS diagnosis: comparison between screen-detected and interval DCIS (DCIS diagnostic period 1999–2004 (screening implemented)). Age was the primary time scale and time since DCIS diagnosis (0–5, 5–10, and ≥10 years) was the secondary time scale. Model 1 was adjusted for period of DCIS diagnosis, DCIS grade, and DCIS treatment (time-varying). Model 2 was adjusted for period of DCIS diagnosis, DCIS grade, DCIS treatment (time-varying), and the occurrence of ipsilateral and contralateral invasive breast cancer (time-varying). (DOCX 20 kb)

## Abbreviations

CI: Confidence interval
DCIS: Ductal carcinoma in situ
HR: Hazard ratio
NCR: Netherlands Cancer Registry
PALGA: The nationwide network and registry of histology and cytopathology in the Netherlands

## References

[1] Esserman LJ, Thompson IM, Reid B (2013). Overdiagnosis and overtreatment in cancer: an opportunity for improvement. JAMA.

[2] Welch HG, Passow HJ (2014). Quantifying the benefits and harms of screening mammography. JAMA Intern Med.

[3] Marmot MG, Altman DG, Cameron DA, Dewar JA, Thompson SG, Wilcox M (2013). The benefits and harms of breast cancer screening: an independent review. Br J Cancer.

[4] Carter JL, Coletti RJ, Harris RP (2015). Quantifying and monitoring overdiagnosis in cancer screening: a systematic review of methods. BMJ.

[5] Barratt A (2015). Overdiagnosis in mammography screening: a 45 year journey from shadowy idea to acknowledged reality. BMJ.

[6] Harding C, Pompei F, Burmistrov D, Welch HG, Abebe R, Wilson R. Breast cancer screening, incidence, and mortality across US counties. JAMA Intern Med. 2015;175(9):1483–9. doi:10.1001/jamainternmed.2015.3043. 10.1001/jamainternmed.2015.3043 26147578

[7] Independent UK Panel on Breast Cancer Screening (2012). The benefits and harms of breast cancer screening: an independent review. Lancet.

[8] Welch HG (2009). Overdiagnosis and mammography screening. BMJ.

[9] Alvarado M, Ozanne E, Esserman L. Overdiagnosis and overtreatment of breast cancer. Am Soc Clin Oncol Educ Book. 2012:e40–5. doi:10.14694/EdBook_AM.2012.32.e40. 10.14694/EdBook_AM.2012.32.301 24451829

[10] Jørgensen KJ, Gøtzsche PC (2009). Overdiagnosis in publicly organised mammography screening programmes: systematic review of incidence trends. BMJ.

[11] Ernster VL, Barclay J, Kerlikowske K, Grady D, Henderson C (1996). Incidence of and treatment for ductal carcinoma in situ of the breast. JAMA.

[12] Ripping TM, Verbeek ALM, Fracheboud J, de Koning HJ, van Ravesteyn NT, Broeders MJM (2015). Overdiagnosis by mammographic screening for breast cancer studied in birth cohorts in The Netherlands. Int J Cancer.

[13] Duffy SW, Dibden A, Michalopoulos D, Offman J, Parmar D, Jenkins J, Collins B, Robson T, Scorfield S, Green K, Hall C, Liao X-H, Ryan M, Johnson F, Stevens G, Kearins O, Sellars S, Patnick J (2016). Screen detection of ductal carcinoma in situ and subsequent incidence of invasive interval breast cancers: a retrospective population-based study. Lancet Oncol.

[14] Mook S, van ’t Veer LJ, Rutgers EJ, Ravdin PM, van de Velde AO, van Leeuwen FE, Visser O, Schmidt MK (2011). Independent prognostic value of screen detection in invasive breast cancer. J Natl Cancer Inst.

[15] Wishart GC, Greenberg DC, Britton PD, Chou P, Brown CH, Purushotham AD, Duffy SW (2008). Screen-detected vs symptomatic breast cancer: is improved survival due to stage migration alone?. Br J Cancer.

[16] Zhang X, Dai H, Liu B, Song F, Chen K (2016). Predictors for local invasive recurrence of ductal carcinoma in situ of the breast: a meta-analysis. Eur J Cancer Prev.

[17] Wang S-Y, Shamliyan T, Virnig BA, Kane R (2011). Tumor characteristics as predictors of local recurrence after treatment of ductal carcinoma in situ: a meta-analysis. Breast Cancer Res Treat.

[18] Falk RS, Hofvind S, Skaane P, Haldorsen T (2011). Second events following ductal carcinoma in situ of the breast: a register-based cohort study. Breast Cancer Res Treat.

[19] Cheung S, Booth ME, Kearins O, Dodwell D (2014). Risk of subsequent invasive breast cancer after a diagnosis of ductal carcinoma in situ (DCIS). Breast..

[20] Casparie M, Tiebosch ATMG, Burger G, Blauwgeers H, van de Pol A, van Krieken JHJM, Meijer GA (2007). Pathology databanking and biobanking in The Netherlands, a central role for PALGA, the nationwide histopathology and cytopathology data network and archive. Cell Oncol.

[21] National Evaluation Team for Breast cancer screening, Fracheboud J, van Luijt PA, Sankkatsing V, Ripping TM, Broeders M, Otten J, van Ineveld BM, Heijnsdijk E, Verbeek A, Holland R, den GJ H, de Bruijn AE, de Koning HJ. National evaluation of breast cancer screening in the Netherlands 1990 – 2011/2012. 2014. http://www.erasmusmc.nl/public-health/publications-collaborations/reports/evaluatie-borstkanker/pdf/?lang=en. Accessed 1 Oct 2016.

[22] de Koning HJ, Fracheboud J, Boer R, Verbeek AL, Collette HJ, Hendriks JH, van Ineveld BM, de Bruyn AE, van der Maas PJ (1995). Nation-wide breast cancer screening in The Netherlands: support for breast-cancer mortality reduction. National Evaluation Team for Breast Cancer Screening (NETB). Int J Cancer.

[23] Fracheboud J, de Koning HJ, Beemsterboer PM, Boer R, Hendriks JH, Verbeek AL, van Ineveld BM, de Bruyn AE, van der Maas PJ (1998). Nation-wide breast cancer screening in The Netherlands: results of initial and subsequent screening 1990-1995. National Evaluation Team for Breast Cancer Screening. Int J Cancer.

[24] Holland R, Peterse JL, Millis RR, Eusebi V, Faverly D, van de Vijver MJ, Zafrani B (1994). Ductal carcinoma in situ: a proposal for a new classification. Semin Diagn Pathol.

[25] Fine JPG, Gray RJ (1999). A proportional hazards model for the subdistribution of a competing risk. J Am Stat Assoc.

[26] Narod SA, Iqbal J, Giannakeas V, Sopik V, Sun P (2015). Breast cancer mortality after a diagnosis of ductal carcinoma in situ. JAMA Oncol..

[27] Silverman SL, Gold DT (2011). Healthy users, healthy adherers, and healthy behaviors?. J Bone Miner Res.

[28] Aarts MJ, Voogd AC, Duijm LEM, Coebergh JWW, Louwman WJ (2011). Socioeconomic inequalities in attending the mass screening for breast cancer in the south of the Netherlands--associations with stage at diagnosis and survival. Breast Cancer Res Treat.

[29] Lee JR, Vogel VG (1995). Who uses screening mammography regularly?. Cancer Epidemiol Biomarkers Prev.

[30] Hofer TP, Katz SJ (1996). Healthy behaviors among women in the United States and Ontario: the effect on use of preventive care. Am J Public Health.

[31] Boekel NB, Schaapveld M, Gietema JA, Rutgers EJT, Versteegh MIM, Visser O, Aleman BMP, van Leeuwen FE (2014). Cardiovascular morbidity and mortality after treatment for ductal carcinoma in situ of the breast. J Natl Cancer Inst.

[32] Ernster VL, Barclay J, Kerlikowske K, Wilkie H, Ballard-Barbash R (2000). Mortality among women with ductal carcinoma in situ of the breast in the population-based surveillance, epidemiology and end results program. Arch Intern Med.

[33] Elshof LE, Schaapveld M, Schmidt MK, Rutgers EJ, van Leeuwen FE, Wesseling J (2016). Subsequent risk of ipsilateral and contralateral invasive breast cancer after treatment for ductal carcinoma in situ: incidence and the effect of radiotherapy in a population-based cohort of 10,090 women. Breast Cancer Res Treat.

[34] Barnes NLP, Dimopoulos N, Williams KE, Howe M, Bundred NJ (2014). The frequency of presentation and clinico-pathological characteristics of symptomatic versus screen detected ductal carcinoma in situ of the breast. Eur J Surg Oncol.

[35] Koh VCY, Lim JCT, Thike AA, Cheok PY, Thu MMM, Tan VKM, Tan BKT, Ong KW, Ho GH, Tan WJ, Tan Y, Salahuddin AS, Busmanis I, Chong APY, Iqbal J, Thilagaratnam S, Wong JSL, Tan PH (2015). Characteristics and behaviour of screen-detected ductal carcinoma in situ of the breast: comparison with symptomatic patients. Breast Cancer Res Treat.

[36] Evans AJ, Pinder SE, Ellis IO, Wilson AR (2001). Screen detected ductal carcinoma in situ (DCIS): overdiagnosis or an obligate precursor of invasive disease?. J Med Screen.

[37] van Luijt PA, Heijnsdijk EAM, Fracheboud J, Overbeek LIH, Broeders MJM, Wesseling J, den Heeten GJ, de Koning HJ (2016). The distribution of ductal carcinoma in situ (DCIS) grade in 4232 women and its impact on overdiagnosis in breast cancer screening. Breast Cancer Res.

[38] Boyd NF, Guo H, Martin LJ, Sun L, Stone J, Fishell E, Jong RA, Hislop G, Chiarelli A, Minkin S, Yaffe MJ (2007). Mammographic density and the risk and detection of breast cancer. N Engl J Med.

[39] Carney PA, Miglioretti DL, Yankaskas BC, Kerlikowske K, Rosenberg R, Rutter CM, Geller BM, Abraham LA, Taplin SH, Dignan M, Cutter G, Ballard-Barbash R (2003). Individual and combined effects of age, breast density, and hormone replacement therapy use on the accuracy of screening mammography. Ann Intern Med.

[40] Shen Y, Yang Y, Inoue LYT, Munsell MF, Miller AB, Berry DA (2005). Role of detection method in predicting breast cancer survival: analysis of randomized screening trials. J Natl Cancer Inst.

[41] Senkus E, Kyriakides S, Ohno S, Penault-Llorca F, Poortmans P, Rutgers E, Zackrisson S, Cardoso F, ESMO Guidelines Committee (2015). Primary breast cancer: ESMO Clinical Practice Guidelines for diagnosis, treatment and follow-up. Ann Oncol.

[42] Vigeland E, Klaasen H, Klingen TA, Hofvind S, Skaane P (2008). Full-field digital mammography compared to screen film mammography in the prevalent round of a population-based screening programme: the Vestfold County Study. Eur Radiol.

[43] Bluekens AMJ, Holland R, Karssemeijer N, Broeders MJM, den GJ H (2012). Comparison of digital screening mammography and screen-film mammography in the early detection of clinically relevant cancers: a multicenter study. Radiology.

[44] van Luijt PA, Fracheboud J, Heijnsdijk EAM, den GJ H, de Koning HJ (2013). National Evaluation Team for Breast Cancer Screening in Netherlands Study Group (NETB). Nation-wide data on screening performance during the transition to digital mammography: observations in 6 million screens. Eur J Cancer.

[45] de Munck L, de Bock GH, Otter R, Reiding D, Broeders MJ, Willemse PH, Siesling S (2016). Digital vs screen-film mammography in population-based breast cancer screening: performance indicators and tumour characteristics of screen-detected and interval cancers. Br J Cancer.

[46] Nederend J, Duijm LEM, Louwman MWJ, Groenewoud JH, Donkers-van Rossum AB, Voogd AC (2012). Impact of transition from analog screening mammography to digital screening mammography on screening outcome in The Netherlands: a population-based study. Ann Oncol.

