# Supplementary material for: The method of detection of ductal carcinoma in situ has no therapeutic implications: results of a population-based cohort study
Source: Breast Cancer Res. 2017 Mar 9;19:26. doi: 10.1186/s13058-017-0819-4 (PMC5343406; doi:10.1186/s13058-017-0819-4)
Supplement: Additional file 1: — Time (days) between a first or subsequent screening examination by the Dutch breast cancer screening program and DCIS diagnosis in women with screen-detected DCIS (DCIS diagnostic period 1989–2004) (DOCX 17 kb) [file 13058_2017_819_MOESM1_ESM.docx]

**Additional file 1. Time between a first or subsequent screening examination by the Dutch breast cancer screening program and DCIS diagnosis in women with screen-detected DCIS**
