# Supplementary material for: The method of detection of ductal carcinoma in situ has no therapeutic implications: results of a population-based cohort study
Source: Breast Cancer Res. 2017 Mar 9;19:26. doi: 10.1186/s13058-017-0819-4 (PMC5343406; doi:10.1186/s13058-017-0819-4)
Supplement: Additional file 2: — Multivariable-adjusted Cox regression analysis of ipsilateral and contralateral invasive breast cancer in women aged 49–75 years at DCIS diagnosis (DCIS diagnostic period 1989–2004). Age was the primary time scale, time since DCIS diagnosis (0–5, 5–10, and ≥10 years) the secondary time scale, and DCIS treatment a time-varying covariable (DOCX 22 kb) [file 13058_2017_819_MOESM2_ESM.docx]

**Additional file 2. Multivariable-adjusted Cox regression analysis for ipsilateral and contralateral invasive breast cancer in women aged 49-75 years at DCIS diagnosis***

|  | Total ipsilateral invasive breast cancer | Person-time, years | HR (95% CI) | p-value |
| --- | --- | --- | --- | --- |
| Method of detection | | |  |  |
| Non-screening-related | 98 | 18710 | ref |  |
| Screen-detected | 231 | 50422 | 0.75 (0.59-0.96) | 0.024 |
| Interval | 34 | 6359 | 1.02 (0.68-1.51) | 0.941 |
| Treatment | |  |  |  |
| Breast conserving surgery with radiotherapy | 88 | 18815 | ref |  |
| Breast conserving surgery alone | 245 | 18367 | 2.51 (1.94-3.26) | <0.001 |
| Mastectomy | 30 | 38309 | 0.15 (0.10-0.23) | <0.001 |
| Year of diagnosis | |  |  |  |
| 1989 - 1998 | 244 | 44070 | ref |  |
| 1999 - 2004 | 119 | 31421 | 0.72 (0.55-0.94) | 0.016 |
| Grade |  |  |  |  |
| 1 | 42 | 5933 | ref |  |
| 2 | 47 | 11152 | 0.91 (0.60-1.38) | 0.651 |
| 3 | 60 | 18803 | 0.88 (0.59-1.31) | 0.528 |
| Unknown | 214 | 39603 | 0.96 (0.67-1.37) | 0.819 |
| Follow-up interval | |  |  |  |
| 0-5 years | 156 | 34180 | ref |  |
| 5-10 years | 154 | 26391 | 1.22 (0.97-1.55) | 0.093 |
| >10 years | 53 | 14920 | 0.69 (0.49-0.98) | 0.040 |
|  | **Total contralateral invasive breast cancer** | **Person-time, years** | **HR (95% CI)** | **p-value** |
| Method of detection | | |  |  |
| Non-screening-related | 105 | 18825 | ref |  |
| Screen-detected | 243 | 50467 | 0.86 (0.67-1.10) | 0.219 |
| Interval | 30 | 6387 | 0.83 (0.54-1.26) | 0.376 |
| Treatment | |  |  |  |
| Breast conserving surgery with radiotherapy | 97 | 18785 | ref |  |
| Breast conserving surgery alone | 106 | 19434 | 1.11 (0.83-1.48) | 0.489 |
| Mastectomy | 175 | 37460 | 0.92 (0.71-1.19) | 0.517 |
| Year of diagnosis | |  |  |  |
| 1989 - 1998 | 209 | 44457 | ref |  |
| 1999 - 2004 | 169 | 31222 | 1.23 (0.94-1.61) | 0.129 |
| Grade |  |  |  |  |
| 1 | 32 | 6005 | ref |  |
| 2 | 64 | 11057 | 1.13 (0.74-1.74) | 0.578 |
| 3 | 91 | 18653 | 0.97 (0.64-1.46) | 0.883 |
| Unknown | 191 | 39964 | 0.98 (0.66-1.47) | 0.930 |
| Follow-up interval | |  |  |  |
| 0-5 years | 154 | 34166 | ref |  |
| 5-10 years | 148 | 26427 | 1.29 (1.01-1.65) | 0.038 |
| >10 years | 76 | 15086 | 1.17 (0.84-1.64) | 0.359 |

* With age as primary time-scale and time since DCIS diagnosis (0-5, 5-10, and ≥10 years) as secondary time-scale.

HR = hazard ratio; CI = confidence interval.
