# Supplementary material for: The method of detection of ductal carcinoma in situ has no therapeutic implications: results of a population-based cohort study
Source: Breast Cancer Res. 2017 Mar 9;19:26. doi: 10.1186/s13058-017-0819-4 (PMC5343406; doi:10.1186/s13058-017-0819-4)
Supplement: Additional file 3: — Multivariable-adjusted Cox regression analysis of overall mortality in women aged 49–75 years at DCIS diagnosis (DCIS diagnostic period 1989–2004). Age was the primary time scale and time since DCIS diagnosis (0–5, 5–10, and ≥10 years) the secondary time scale. Model 1 was adjusted for period of DCIS diagnosis, DCIS grade, and DCIS treatment (time-varying). Model 2 was adjusted for period of DCIS diagnosis, DCIS grade, DCIS treatment (time-varying), and the occurrence of ipsilateral and contralateral invasive breast cancer (time-varying) (DOCX 21 kb) [file 13058_2017_819_MOESM3_ESM.docx]

**Additional file 3. Multivariable-adjusted Cox regression analysis for overall mortality in women aged 49-75 years at DCIS diagnosis***

|  | Total deaths | Person-time, years | HR** (95% CI) | p-value | HR*** (95% CI) | p-value |
| --- | --- | --- | --- | --- | --- | --- |
| Method of detection |  |  |  |  |  |  |
| Non-screening-related | 398 | 19361 | ref |  | ref |  |
| Screen-detected | 594 | 51740 | 0.85 (0.73-0.98) | 0.028 | 0.86 (0.75-1.00) | 0.054 |
| Interval | 68 | 6570 | 0.73 (0.56-0.96) | 0.025 | 0.73 (0.56-0.96) | 0.022 |
| Treatment |  |  |  |  |  |  |
| Breast conserving surgery with radiotherapy | 193 | 19191 | ref |  | ref |  |
| Breast conserving surgery alone | 329 | 20013 | 1.31 (1.09-1.58) | 0.004 | 1.14 (0.95-1.38) | 0.165 |
| Mastectomy | 538 | 38467 | 1.03 (0.87-1.23) | 0.706 | 1.10 (0.92-1.30) | 0.299 |
| Year of diagnosis |  |  |  |  |  |  |
| 1989 - 1998 | 751 | 45779 | ref |  | ref |  |
| 1999 - 2004 | 309 | 31892 | 0.79 (0.66-0.94) | 0.009 | 0.80 (0.66-0.95) | 0.012 |
| Grade |  |  |  |  |  |  |
| 1 | 78 | 6148 | ref |  | ref |  |
| 2 | 124 | 11370 | 1.00 (0.75-1.33) | 0.984 | 1.01 (0.76-1.35) | 0.941 |
| 3 | 190 | 19083 | 0.95 (0.72-1.24) | 0.681 | 0.96 (0.74-1.26) | 0.794 |
| Unknown | 668 | 41070 | 0.97 (0.75-1.25) | 0.807 | 0.98 (0.76-1.26) | 0.887 |
| Follow-up interval |  |  |  |  |  |  |
| 0-5 years | 285 | 34507 | ref |  | ref |  |
| 5-10 years | 385 | 27306 | 1.09 (0.93-1.29) | 0.273 | 1.03 (0.88-1.21) | 0.732 |
| >10 years | 390 | 15858 | 1.04 (0.86-1.26) | 0.713 | 0.94 (0.77-1.14) | 0.515 |
| Ipsilateral invasive breast cancer |  |  |  |  |  |  |
| No | 945 | 75491 | NA | NA | ref |  |
| Yes | 115 | 2180 | NA | NA | 2.92 (2.36-3.61) | <0.001 |
| Contralateral invasive breast cancer |  |  |  |  |  |  |
| No | 992 | 75679 | NA | NA | ref |  |
| Yes | 68 | 1992 | NA | NA | 1.87 (1.46-2.40) | <0.001 |

* With age as primary time-scale and time since DCIS diagnosis (0-5, 5-10, and ≥10 years) as secondary time-scale.

** Adjusted for period of DCIS diagnosis, DCIS grade and DCIS treatment (time-varying).

*** Adjusted for period of DCIS diagnosis, DCIS grade, DCIS treatment (time-varying) and the occurrence of ipsilateral and contralateral invasive breast cancer (time-varying).

HR = hazard ratio; CI = confidence interval; NA = not applicable.
