# Supplementary material for: The method of detection of ductal carcinoma in situ has no therapeutic implications: results of a population-based cohort study
Source: Breast Cancer Res. 2017 Mar 9;19:26. doi: 10.1186/s13058-017-0819-4 (PMC5343406; doi:10.1186/s13058-017-0819-4)
Supplement: Additional file 4: — Kaplan-Meier curves for all-cause mortality by method of detection for patients aged 49–59 years at DCIS diagnosis (a), patients aged 60–69 years at DCIS diagnosis (b), and patients aged 70–75 years (c) at DCIS diagnosis (DCIS diagnostic period 1989–2004). P values based on Cox proportional hazards regression with time since DCIS diagnosis as the primary time scale, adjusted for age (continuous). (DOCX 52 kb) [file 13058_2017_819_MOESM4_ESM.docx]

**Additional file 4. Kaplan-Meier curves for all-cause mortality by method of detection for A) patients aged 49-59 years at DCIS diagnosis, B) patients aged 60-69 years at DCIS diagnosis, and C) patients aged 70-75 years at DCIS diagnosis. P-values based on Cox proportional hazards regression with time since DCIS diagnosis as primary time-scale, adjusted for age (continuous).**

Overall *P* =0.598

Screen-detected vs non-screening-related *P* =0.921

Interval vs non-screening-related *P* =0.336

A****

Overall *P* =<0.001

Screen-detected vs non-screening-related *P* =<0.001

Interval vs non-screening-related *P* =0.011

B****

Overall *P* =0.562

Screen-detected vs non-screening-related *P* =0.325

Interval vs non-screening-related *P* =0.491

C
