# Supplementary material for: The method of detection of ductal carcinoma in situ has no therapeutic implications: results of a population-based cohort study
Source: Breast Cancer Res. 2017 Mar 9;19:26. doi: 10.1186/s13058-017-0819-4 (PMC5343406; doi:10.1186/s13058-017-0819-4)
Supplement: Additional file 5: — Multivariable-adjusted Cox regression analysis of ipsilateral and contralateral invasive breast cancer in women aged 49–75 years at DCIS diagnosis: comparison between screen-detected and interval DCIS (DCIS diagnostic period 1999–2004 (screening implemented)). Age was the primary time scale and time since DCIS diagnosis (0–5, 5–10, and ≥10 years) the secondary time-scale. (DOCX 21 kb) [file 13058_2017_819_MOESM5_ESM.docx]

**Additional file 5. Multivariable-adjusted Cox regression analysis for ipsilateral and contralateral invasive breast cancer in women aged 49-75 years at DCIS diagnosis – comparison between screen-detected and interval DCIS, *1999-2004****

|  | Total ipsilateral invasive breast cancer | Person-time, years | HR (95% CI) | p-value |
| --- | --- | --- | --- | --- |
| Method of detection |  |  |  |  |
| Screen-detected | 82 | 23426 | ref |  |
| Interval | 19 | 3584 | 1.64 (0.99-2.72) | 0.056 |
| Treatment |  |  |  |  |
| Breast-conserving surgery with radiotherapy | 41 | 10738 | ref |  |
| Breast-conserving surgery alone | 55 | 4712 | 3.01 (1.97-4.59) | <0.001 |
| Mastectomy | 5 | 11560 | 0.11 (0.04-0.27) | <0.001 |
| Grade |  |  |  |  |
| 1 | 18 | 3508 | ref |  |
| 2 | 28 | 7065 | 1.31 (0.71-2.40) | 0.386 |
| 3 | 32 | 11923 | 1.16 (0.64-2.13) | 0.620 |
| Unknown | 23 | 4514 | 1.28 (0.69-2.38) | 0.435 |
| Follow-up interval |  |  |  |  |
| 0-5 years | 45 | 15859 | ref |  |
| 5-10 years | 54 | 10409 | 1.76 (1.15-2.68) | 0.009 |
| >10 years | 2 | 742 | 0.80 (0.19-3.36) | 0.764 |
|  |  |  |  |  |
|  | **Total contralateral invasive breast cancer** | **Person-time, years** | **HR (95% CI)** | **p-value** |
| Method of detection |  |  |  |  |
| Screen-detected | 118 | 23275 | ref |  |
| Interval | 18 | 3586 | 1.01 (0.61-1.67) | 0.958 |
| Treatment |  |  |  |  |
| Breast-conserving surgery with radiotherapy | 59 | 10668 | ref |  |
| Breast-conserving surgery alone | 28 | 4874 | 1.06 (0.67-1.68) | 0.812 |
| Mastectomy | 49 | 11318 | 0.81 (0.55-1.81) | 0.271 |
| Grade |  |  |  |  |
| 1 | 20 | 3509 | ref |  |
| 2 | 47 | 6967 | 1.23 (0.72-2.11) | 0.447 |
| 3 | 51 | 11821 | 0.80 (0.47-1.37) | 0.409 |
| Unknown | 18 | 4565 | 0.71 (0.38-1.35) | 0.302 |
| Follow-up interval |  |  |  |  |
| 0-5 years | 67 | 15797 | ref |  |
| 5-10 years | 68 | 10318 | 1.61 (1.12-2.31) | 0.010 |
| >10 years | 1 | 745 | 0.35 (0.05-2.53) | 0.297 |

* With age as primary time-scale and time since DCIS diagnosis (0-5, 5-10, and ≥10 years) as secondary time-scale.

HR = hazard ratio; CI = confidence interval.
