# Supplementary material for: The method of detection of ductal carcinoma in situ has no therapeutic implications: results of a population-based cohort study
Source: Breast Cancer Res. 2017 Mar 9;19:26. doi: 10.1186/s13058-017-0819-4 (PMC5343406; doi:10.1186/s13058-017-0819-4)
Supplement: Additional file 6: — Multivariable-adjusted Cox regression analysis of overall mortality in women aged 49–75 years at DCIS diagnosis: comparison between screen-detected and interval DCIS (DCIS diagnostic period 1999–2004 (screening implemented)). Age was the primary time scale and time since DCIS diagnosis (0–5, 5–10, and ≥10 years) was the secondary time scale. Model 1 was adjusted for period of DCIS diagnosis, DCIS grade, and DCIS treatment (time-varying). Model 2 was adjusted for period of DCIS diagnosis, DCIS grade, DCIS treatment (time-varying), and the occurrence of ipsilateral and contralateral invasive breast cancer (time-varying). (DOCX 20 kb) [file 13058_2017_819_MOESM6_ESM.docx]

**Additional file 6. Multivariable-adjusted Cox regression analysis for overall mortality in women aged 49-75 years at DCIS diagnosis – comparison between screen-detected and interval DCIS, *1999-2004****

|  | Total deaths | Person-time, years | HR** (95% CI) | p-value | HR*** (95% CI) | p-value |
| --- | --- | --- | --- | --- | --- | --- |
| Method of detection |  |  |  |  |  |  |
| Screen-detected | 224 | 23747 | ref |  | ref |  |
| Interval | 38 | 3664 | 1.00 (0.70-1.41) | 0.978 | 0.95 (0.67-1.35) | 0.773 |
| Treatment |  |  |  |  |  |  |
| Breast-conserving surgery with radiotherapy | 91 | 10869 | ref |  | ref |  |
| Breast-conserving surgery alone | 64 | 4968 | 1.43 (1.03-1.99) | 0.034 | 1.29 (0.92-1.80) | 0.146 |
| Mastectomy | 107 | 11574 | 1.09 (0.82-1.44) | 0.559 | 1.12 (0.84-1.48) | 0.435 |
| Grade |  |  |  |  |  |  |
| 1 | 39 | 3575 | ref |  | ref |  |
| 2 | 76 | 7177 | 1.08 (0.73-1.61) | 0.689 | 1.08 (0.73-1.25) | 0.695 |
| 3 | 101 | 12037 | 0.90 (0.61-1.33) | 0.589 | 0.91 (0.61-1.33) | 0.615 |
| Unknown | 46 | 4622 | 0.94 (0.61-1.44) | 0.775 | 0.94 (0.61-1.45) | 0.788 |
| Follow-up interval |  |  |  |  |  |  |
| 0-5 years | 123 | 15949 | ref |  | ref |  |
| 5-10 years | 131 | 10668 | 1.01 (0.77-1.32) | 0.938 | 0.96 (0.73-1.25) | 0.741 |
| >10 years | 8 | 793 | 0.64 (0.31-1.35) | 0.242 | 0.53 (0.25-1.12) | 0.098 |
| Ipsilateral invasive breast cancer |  |  |  |  |  |  |
| No | 245 | 27009 | NA | NA | ref |  |
| Yes | 17 | 401 | NA | NA | 3.32 (1.96-5.63) | <0.001 |
| Contralateral invasive breast cancer |  |  |  |  |  |  |
| No | 252 | 26861 | NA | NA | ref |  |
| Yes | 10 | 550 | NA | NA | 1.79 (0.94-3.38) | 0.074 |

* With age as primary time-scale and time since DCIS diagnosis (0-5, 5-10, and ≥10 years) as secondary time-scale.

** Adjusted for period of DCIS diagnosis, DCIS grade and DCIS treatment (time-varying).

*** Adjusted for period of DCIS diagnosis, DCIS grade, DCIS treatment (time-varying) and the occurrence of ipsilateral and contralateral invasive breast cancer (time-varying).

HR = hazard ratio; CI = confidence interval; NA = not applicable.
